# Supplemental invasion of *Salmonella* from the perspective of *Salmonella enterica* serovars Kentucky and Typhimurium

**DOI:** 10.1186/s12866-017-0989-3

**Published:** 2017-04-05

**Authors:** Kevin Howe, Sanaz Salehi, R. Hartford Bailey, John P. Brooks, Robert Wills, Mark L. Lawrence, Attila Karsi

**Affiliations:** 1 Department of Pathobiology and Population Medicine, College of Veterinary Medicine, Mississippi State University, Mississippi State, Mississippi State, MS USA; 2 USDA-ARS, Genetics and Precision Agriculture Unit, Mississippi State, MS USA; 3 Department of Basic Sciences, College of Veterinary Medicine, Mississippi State University, Mississippi State, MS USA

**Keywords:** *Salmonella*, Kentucky, Typhimurium, Type III Secretion System, Invasion, Internalization, Host Tropism

## Abstract

**Background:**

Critical to the development of Salmonellosis in humans is the interaction of the bacterium with the epithelial lining of the gastrointestinal tract. Traditional scientific reasoning held type III secretion system (T3SS) as the virulence factor responsible for bacterial invasion. In this study, field-isolated *Salmonella enterica* serovar Kentucky and a known human pathogen *Salmonella enterica* serovar Typhimurium were mutated and evaluated for the invasion of human colorectal adenocarcinoma epithelial cells.

**Results:**

*S. enterica* serovar Kentucky was shown to actively invade a eukaryotic monolayer, though at a rate that was significantly lower than Typhimurium. Additionally, strains mutated for T3SS formation were less invasive than the wild-type strains, but the decrease in invasion was not significant in Kentucky.

**Conclusions:**

Strains mutated for T3SS formation were able to initiate invasion of the eukaryotic monolayer to varying degrees based on strain, In the case of Kentucky, the mutated strain initiated invasion at a level that was not significantly different from the wild-type strain. A different result was observed for Typhimurium as the mutation significantly lowered the rate of invasion in comparison to the wild-type strain.

## Background

The interaction between *Salmonella* and host cells has been shaped by intimate coexistence, leading to a rather balanced interaction that allows bacterial replication while preventing excessive harm unless the host is compromised. The various serotypes of *Salmonella enterica* cause different diseases, ranging from self-limiting gastroenteritis to systemic illness such as typhoid fever. Central to the pathogenesis of *Salmonella* is the invasion of the gastrointestinal epithelium tissue. To accomplish this task, the bacteria have evolved virulence traits to adapt to their host that enables the organisms to induce their own internalization by nonphagocytic cells to successfully survive and replicate intracellularly [1]. The most extensively studied mechanism of invasion involves a multiprotein complex termed the type III secretion system (T3SS). Until recently, it was accepted that *Salmonella* invasion of eukaryotic cells required only T3SS encoded in the *Salmonella* Pathogenicity Island-1 (SPI1). However, recent studies have shown that *Salmonella* can cause infection in a manner independent of T3SS [2, 3]. This data sheds light on a new paradigm that slightly contrasts with our traditional understanding, and lends itself to the possibility of unknown entry routes that may be specific to the serotype, the host, and the cell type considered, potentially affecting the outcome of different *Salmonella*-induced diseases.

*Salmonella* entry into host cells depends on the function of the actin cytoskeleton as the addition of drugs that interfere with actin dynamics effectively block bacterial internalization [4]. An emerging perspective on *Salmonella* speculates infection of gastrointestinal epithelium may occur through multiple pathways, which contrasts with the traditional essential role of T3SS in bacterial invasion of host cells. Although numerous studies demonstrate a role of T3SS in host infection, recent studies using bovine, chicken, murine, and human models to determine the function of SPI1 suggest *Salmonella* can cause infection in a manner independent of T3SS [2, 3, 5, 6]. Radtke et al. demonstrated *Salmonella enterica* serovar Typhimurium was capable of active invasion of a 3-D model of human colonic epithelium cells without the expression of T3SS genes [6]. Desin et al. observed a *Salmonella enterica* serovar Enteritidis strain mutated for SPI1 expression displayed invasion of polarized human intestinal epithelial cells (Caco-2) and chicken intestinal tissue explants in a reduced capacity in comparison to the wild-type strain [5]. The same study orally challenged 1-week-old chicks with the wild-type or the SPI1 mutant strain, and no difference in cecal colonization was observed thus suggesting infection mechanisms correspond with chicken maturity and infection in this situation may not be exclusive to T3SS [5]. Similar findings were observed by Morgan et al., in which disruption of T3SS caused only minor reduction in *Salmonella enterica* serovar Typhimurium colonization of chicks [7]. The type of interaction between *Salmonella* and its host leading to infection is sophisticated and dynamic, dependent on the serovar, cell-type considered, and host. Evidence in the literature suggests additional mechanisms may act in a complementary role to T3SS to facilitate and modify the initiation of eukaryotic cell entry.

Spanning the last several decades, there have been significant shifts in *Salmonella* populations associated with poultry and human infections. Among the leading serotypes associated with human infections over the last quarter century, the widespread prevalence of *Salmonella enterica* serovar Enteritidis among poultry developed into a significant problem in the commercial poultry industry [8, 9] until the National Poultry Improvement Plan (NPIP) began targeting Enteritidis for eradication in eggs in 1989 and meats in 1994 [10]. The ecological niche in poultry created by the reduction of Enteritidis may have been filled by *Salmonella enterica* serovar Heidelberg and *Salmonella enterica* serovar Kentucky due to differences in surface antigens [11]. Initially isolated from a chick in the U.S. in 1937 [12], *S*. Kentucky has a close association with poultry and poultry products and recently has been the most frequently isolated serotype [13–16]. The practice of antibiotic use in agriculture, especially in the United States, not only to treat and prevent disease but also to promote growth [17] may have contributed to the ascension and dissemination of *S*. Kentucky. Of interest, multidrug resistant variants of *S*. Kentucky have been identified at a high frequency from chickens in the United States [18] and Ireland [19].

In light of recent scientific findings indicating *Salmonella enterica* infection apart from the T3SS-mediated pathway, it appears that a gap in the knowledge exists to characterize the components that are able to continue to facilitate infection. Additionally, the factors that specify host range for *S*. Kentucky are not completely understood, and it remains unclear why *S*. Kentucky is not highly virulent to the human population. In this study, field-isolated *Salmonella enterica* serovar Kentucky and *Salmonella enterica* serovar Typhimurium were mutated to disrupt T3SS expression. The wild-type and mutation strains were evaluated for the invasion of human colorectal adenocarcinoma epithelial cell line modeled after a gentamicin protection assay.

## Methods

### Bacterial strains and plasmids

*Salmonella enterica* serovar Kentucky and *Salmonella enterica* serovar Typhimurium (hereafter referred to as Kentucky and Typhimurium, respectively) were isolated from field studies as part of previous work to isolate and catalog *Salmonella* specimens from the poultry processing continuum [20]. Plasmids pKD46, pKD3, and pCP20 were purchased from Coli Genetic Stock Center at Yale University (New Haven, CT).

### Construction of SPI deletion mutations

**Fig. 1:**
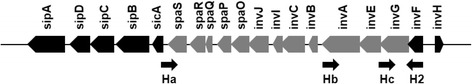
Orientation of mutations in SPI1. The 3′ region of *Salmonella* Pathogenicity Island I. Three deletion mutation events were designed to target structural genes between H*a, b, c* and H2 and are designated as gray arrows. The 40-nt homology extensions are designated Ha, Hb, Hc, and H2

Three deletion mutants targeting structural genes (*invG, invAEG,* and *spaSRQPO*-*invJICBAEG*) were created within SPI1 in Kentucky and Typhimurium following a method developed by Datsenko and Wanner [21]. Briefly, phage λ Red recombinase encoded on plasmid pKD46 replaced a chromosomal sequence with a PCR fragment that has homologous regions adjacent to the chromosomal sequence. The 40-nt homologous regions were designed from *Salmonella enterica* Kentucky genome shotgun sequence (GenBank accession no. ABAK02000001.1) and *Salmonella enterica* Typhimurium str. SL1344 (NCBI Reference Sequence: NC_016810.1). The 1.1-kbp PCR fragment was generated using the 40-nt homologous extensions as primers (Table 1) from template pKD3 (Fig. 1).

**Table 1:** Primer Sequences

Primer	Sequence	Length
CVM29188.01F	TCATTTAATTGCCTCCTGACCTCTATCCAGATAAACACGAgtgtaggctggagctgcttc	60
CVM29188.02F	TTATATTGTTTTTATAACATTCACTGACTTGCTATCTGCTgtgtaggctggagctgcttc	60
CVM29188.03F	TCAATGCCGTACCTCGTTTTCTTGTGGCTGAATAACGTCTgtgtaggctggagctgcttc	60
CVM29188.01R	GCGGAAATTATCAAATATTATTCAATTGGCAGACAAATGAcatatgaatatcctccttag	60

The CVM29188.01F, CVM29188.02F, and CVM29188.03F primers harbor 40 nucleotides that bind upstream of the *invG* gene, *invA* gene, and *spaS* gene, respectively, and 20 nucleotides (in lowercase) that correspond to P1. CVM29188.01R harbors 40 nucleotides that bind downstream of *invG* gene and 20 nucleotides (in lowercase) that correspond to P2

The amplified PCR fragment from plasmid pKD3 encodes a selectable antibiotic resistance gene, chloramphenicol acetyltransferase (*cat*). Plasmid pKD46 transformants containing the PCR fragment after electroporation were selected for successful recombination events within the chromosome using chloramphenicol (Cm) antibiotic pressure (10 μg/mL). Following selection, the resistance gene was eliminated using helper plasmid pCP20, which targets specific FRT (FLP recognition target) sites flanking the resistance gene for recombination [22]. Several attempts to construct complemented Kentucky and Typhimurium Δ*invG* strains were not successful.

### Growth kinetics

Due to limited resources, experiments to determine effects of T3SS mutation on phenotype were conducted using one representative mutant (*invG*). Growth analysis was conducted for wild-type Kentucky and Typhimurium, and their corresponding mutation strains Δ*invG* to determine if bacteria replication was affected by the mutation. Separate cultures were prepared by inoculating fresh LB media with one colony of each serotype and strain and incubated for approximately 16 h at 37 °C with agitation at 200 rpm. Following incubation, OD_600_ values for each culture were adjusted to 1.00 in fresh LB media. Triple technical replicates were prepared for each sample by diluting the culture 1/1000 in fresh LB media and incubated at 37 °C with agitation at 200 rpm. At scheduled time points, starting at 0 h and occurring every hour to 14 h, OD_600_ was measured for each sample. The average OD_600_ values from triple technical replicates for each serotype and strain were plotted against time (m) to create a growth curve depicting bacteria replication as a function of time.

Further growth analysis was conducted to establish viable bacteria number estimates in relation to OD_600_ values. Separate cultures were prepared by inoculating fresh LB media with one colony of each serotype and strain and incubated for approximately 16 h at 37 °C with agitation at 200 rpm. Following incubation, OD_600_ values for each culture were adjusted to 1.00 in fresh LB media. Quadruple technical replicates were prepared for each sample by diluting the culture 1/1000 in fresh LB media and incubated at 37 °C with agitation at 200 rpm. At time point 0 h, an aliquot from each culture was obtained and diluted 10^−3^ in 0.01 M phosphate buffered saline (PBS) (Sigma-Aldrich, St. Louis, MO), and 25 μL aliquots were spread on LB agar to enumerate colony forming units (CFU). Following 6 h period, an aliquot of each sample was removed and diluted 10^−5^ and 10^−6^ in 0.01 M PBS, and 25 μL aliquots were spread on LB agar to enumerate CFU. The previous step was repeated for a successive 6 h period and data collected as previously outlined. Data from the quadruple technical replicates at each time point was gathered to generate an XY scatter plot for each serotype and strain to predict bacteria number in relation to OD_600_. The data was statistically analyzed using linear regression model (PROC REG) SAS for Windows v9.3. All statistical comparisons were two-sided using *P* ≤ 0.05 as the significance level.

### Cell culture

The aim of this study was to investigate the potential risk of foodborne illness in people from *S. enterica* serovars Typhimurium and Kentucky. Thus, human colorectal adenocarcinoma epithelial Caco-2 cells (ATCC HTB-37; ATCC, Manassas, VA) were used. The cells were grown in Eagle’s Minimum Essential Medium (EMEM) (ATCC, Manassas, VA), supplemented with 20% fetal bovine serum (FBS) (Access, Vista, CA) and 2 mM L-glutamine (EMEM-FBS), and maintained at 37 °C in 5% CO_2_ and 95% air, in 75 cm^2^ flasks, unless stated otherwise. Trypsin-treated cells were seeded into 12-well tissue culture plates (Becton and Dickinson and Company, Franklin Lakes, NJ) at approximately 1 × 10^4^ cells per well and grown at 37 °C and 5% CO_2_. Cells on average required 2–5 d to reach 95–100% confluent monolayers (approximately 5 × 10^5^ cells/well). Cell media was exchanged every 2–3 d. Cell number was determined by hemocytometer counts.

### Bacterial infection of Caco-2 cell monolayers

To determine the rate of invasion of Caco-2 cells by Kentucky, Typhimurium, and their corresponding mutant strains, a gentamicin protection assay was performed as described previously [23, 24], with modifications. Briefly, fresh LB media was inoculated with one colony (Kentucky, Kentucky [Δ*invG*], Typhimurium, Typhimurium [Δ*invG*]) and incubated for approximately 16 h at 37 °C with agitation at 200 rpm. Cultures were adjusted to OD_600_ = 0.8 and then diluted 1/1000 in pre-warmed complete cell media to achieve a predetermined bacteria number and transferred to Caco-2 cell monolayers at a multiplicity of infection (MOI) 1:1. Tissue culture plates were briefly centrifuged at 400 rpm for approximately 1 m to bring bacteria into close proximity with the cell monolayer before incubation for 1 h at 37 °C. Removal of cell media with bacteria preceded 2 washes with 0.01 M PBS. Pre-warmed cell media supplemented with gentamicin (200 μg/mL; Gibco) was added to eliminate extracellular bacteria during incubation for 1 h at 37 °C. Cell media solution was removed and kept to be spread (100 μL) on nonselective agar to confirm the antibiotic action. Monolayers were washed twice with 0.01 M PBS, then 500 μL cold Triton X-100 (0.1% *v*/v 0.01 M PBS) was added to lyse cells. The cell suspension was collected, diluted 10^−1^ or 10^−2^, and spread onto nonselective agar in 100 μL aliquots. Viable bacteria were enumerated and adjusted to estimate CFU/mL. Each strain consisted of eight replicates for each trial, and the assay was repeated for three separate trials. The data was statistically analyzed using mixed linear model (PROC MIXED) SAS for Windows v9.3. Strains was the fixed effect, and random effects were trial and trial (strain). The least square means were used to determine statistically significant differences between strains with respect to the mean CFU/mL. All statistical comparisons were two-sided using *P* ≤ 0.05 as the significance level.

## Results

### Construction of SPI deletion mutations

Three sites within SPI1 were targeted for deletion to disrupt T3SS function in Kentucky and Typhimurium. PCR products were generated from pairs of 60-nt primers that included 40-nt homology extensions and 20-nt priming sequences complementary to plasmid pKD3 as a template (Table 2). The PCR products were purified and then transformed into bacteria carrying the λ Red helper plasmid as described previously to create three separate deletion events within the structural genes for T3SS. The mutations were precisely targeted for *invG, invAEG*, and *spaSRQPO*-*invJICBAEG* (Fig. 1).

**Table 2:** Gene disruptions using λ red recombinase

Mutation	Homology Extensions	Priming Sites
ΔSPI1(*invG*)1705	40 nt; Ha: 2,345,733; H2: 2,347,418	P1; P2
ΔSPI1(*invA*-*invG*)4902	40 nt; Hb: 2,342,536; H2: 2,347,418	P1; P2
ΔSPI1(*spaS*-*invG*)11,769	40 nt; Hc: 2,335,669; H2: 2,347,418	P1; P2

All genetic disruptions were made in pKD46 transformants of BW25113. Extension lengths are given first. Numerals identify the 3′ nucleotide of the extension in the Kentucky genome shotgun sequence (GenBank accession no. ABAK02000001.1). One primer had the H*x* extension and the 3′ sequence for priming site 1 (P1). The other primer had the H2 extension and the 3′ sequence for the complement of priming site 2 (P2). Priming sites P1 and P2 associated with plasmid pKD3 [21]

Mutations of this nature interrupt the proper formation of the basal structure that anchors the needle to the bacterial membranes and disrupts proper T3SS function. The outer membrane ring of the basal structure is formed by the integral membrane protein *invG* [25–27]. The export apparatus complex serves as a platform for the assembly of the basal structure and is composed of highly conserved integral membrane proteins: *spaS*, *spaR*, *spaQ*, *spaP*, and *invA* [28]. Protein *spaS* has an additional function to regulate secretion specificity of the virulence effectors through the base [29].

Cm resistance (10 μg/mL) phenotype indicated successful mutations. Antibiotic resistance gene was eliminated by FLP recombinase, expressed from helper plasmid pCP20, which targets the repeated FRT (FLP recognition target) sites flanking the resistance gene. Elimination of the antibiotic resistance gene leaves an 85-nt scar in place of the deleted gene(s) that has stop codons in all six reading frames [21], thereby creating nonpolar gene deletions. Downstream gene expression can continue due to a ribosome binding site and start codon that is encoded in the scar sequence [21].

All gene deletion mutations were verified by PCR tests using locus-specific primers and *cat*-specific primers revealing that all strains had new junction and locus-specific fragments of the predicted sizes. Following the elimination of the resistance gene, PCR test with locus-specific primers indicated the deletion mutants produced the expected junction fragments and verified mutations occurred in the designed location.

### Growth kinetics

Growth analysis was conducted for Kentucky, Typhimurium, and the corresponding mutation strains to determine if bacteria replication was affected by the deletion mutation. Bacteria cultures were adjusted to equal concentration, diluted 1/1000 in media and OD_600_ values were determined from 0 h to 14 h at every hour of incubation at 37 °C at 200 rpm. The OD_600_ values were averaged from quadruple technical replicates and plotted as a function of time (m) for each serotype and strain. Deletion mutations created within SPI1 were not identified as affecting bacterial replication by Kentucky or Typhimurium in relation to their corresponding wild-type strains.

**Fig. 2:**
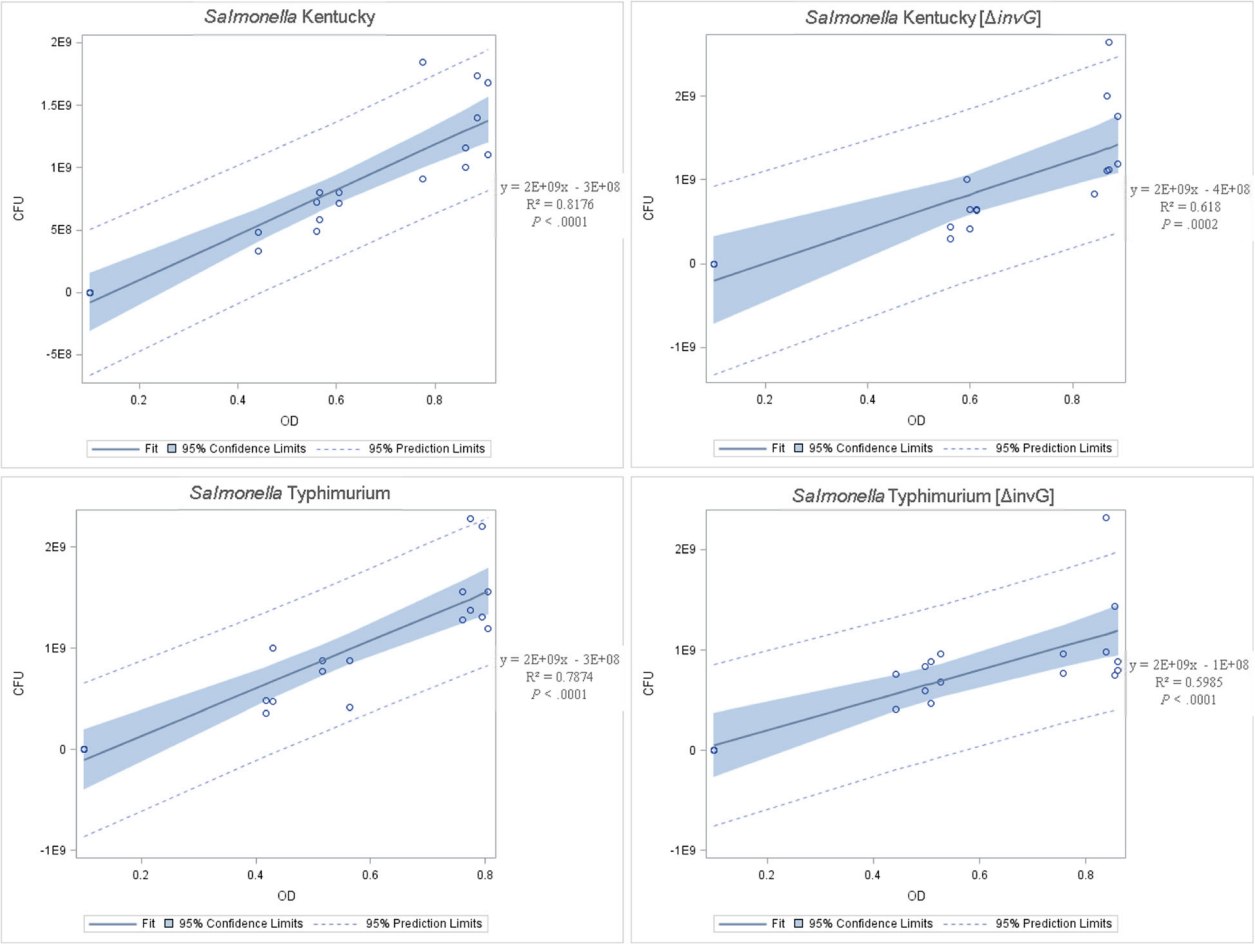
Fit plot for colony forming units (CFU)/mL. At each time point, cultures were diluted either 10^−3^ or 10^−5^ and 10^−6^ in 0.01 M PBS, and 25 μL of bacteria suspension were spread on LB agar. Colony forming units (CFU) from each dilution were enumerated and adjusted to CFU/mL. CFU/mL along the y-axis was plotted as a function of OD_600_ on the x-axis in XY scatter plot to predict CFU relative to OD_600_. The regression equations, R-squared value, and *P*-value were determined for each serotype and strain

Further growth analysis was conducted to confirm viable bacteria number relative to OD_600_ values for each serotype and strain. At time point 0 h, an aliquot of each culture was diluted 10^−3^ in 0.01 M PBS, and at time points 6 h and 12 h, cultures were diluted 10^−5^ and 10^−6^ in 0.01 M PBS, and 25 μL were spread on LB agar. Colony forming units were counted and adjusted to CFU/mL from four technical replicates for each serotype and strain at 0 h, 6 h, and 12 h. For all serotypes and strains, the model used for linear regression analysis indicated a significant portion of the variation in CFU/mL was due to OD_600_. The statistical analysis, including regression equation, R-squared value, *P*-value, for each serotype and strain follows (Fig. 2).

### Invasion rates of wild-type and mutant *S*. Kentucky and *S*. Typhimurium

As wild-type Typhimurium has adapted to humans as a host, a relatively high rate of invasion, approximately 185,625 CFU/mL, in comparison to Kentucky was expected. The invasion rate for wild-type Kentucky was significantly less in comparison to wild-type Typhimurium, approximately 3921 CFU/mL. Though Kentucky is currently the most frequently isolated serotype in commercial poultry, Kentucky is less commonly associated with human disease. Kentucky was not among the 20 most frequently reported serotypes listed for the United States in 2011 [30]. Based on a review of the recent scientific literature, to our knowledge, this is the first study to demonstrate *in vitro* invasion of a human cell line by Kentucky.

**Fig. 3:**
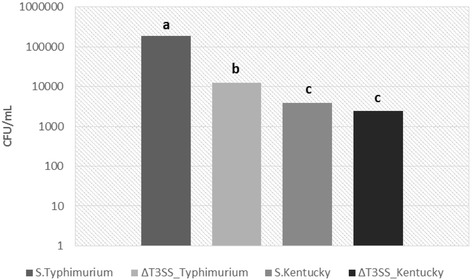
Comparison of rate of invasion of Caco-2 cell monolayer. Strains are denoted along the *x*-axis. Bacteria number are expressed along the *y*-axis and estimated per mL for 1 h invasion period. Strains with the same letter are not significantly different at *P* ≤ 0.05

The deletion mutation (Δ*invG*) of the structural genes associated with T3SS decreased the rate of invasion for both serotypes in comparison to their wild-type strain. Human cells are typically not the specified host for Kentucky, and so the invasion rate for the wild-type strain is limited (3921 CFU/mL). The loss of T3SS function reduced invasion 38.1% from the wild-type to 2425 CFU/mL (Fig. 3), which was not significant (*P* ≤ 0.05). Invasion by Typhimurium was significantly affected by the disruption of T3SS function, decreasing invasion 85.1% from the wild-type to 12,445 CFU/mL. This reduction of invasion rate resulted from the inactivation of T3SS function, which signifies the prominent role this multi-protein complex system contributes to facilitating invasion, a finding that corresponds with previous scientific data concerning the role of T3SS [1, 5, 31, 32]. Active invasion of epithelial cells by the mutation strains represents possible alternative mechanisms of invasion that may act independently and/or in a supplemental role to T3SS.

## Discussion

The frequent isolation of Kentucky serotypes in commercial poultry, especially MDR strains, is significant from a public health standpoint. Angulo et al. suggest that resistance reservoirs in food animals can possibly promote the spread of resistance determinants among bacteria and lead to antimicrobial-resistant infections in humans [33]. Within the last decade, a MDR isolate of Kentucky displaying high-level resistance to ciprofloxacin was disseminated internationally with poultry suspected as the major vehicle for infection [34]. The strain identified as ST198-X1 as CIP^R^ infected nearly 500 patients in France, England and Wales, Denmark, and the United States during 2002–2008. Kentucky isolates have been reported in Europe from travelers returning from northern Africa [35, 36] and more recently, Kentucky isolates with similar macrorestriction patterns to the isolates observed in Europe and Africa have been reported in Canada [37]. A majority of the *S*. Kentucky strains identified were ciprofloxacin-resistant [35–37], and many of the strains contained *Salmonella* genomic island 1 variants [37], resulting in treatment failure with traditional antimicrobials against nontyphoidal *Salmonella* infections due to high level of resistance.

Certain genetic factors have been hypothesized to be responsible for the emergence of *S*. Kentucky in poultry. In a comprehensive study of *Salmonella enterica* serotypes, *S*. Kentucky was shown to have a slight growth advantage over other serotypes in mildly acid environments (pH = 5.5), which may be beneficial in the mildly acidic environment of the cecum [38]. However, studies examining the colonization factors contributing to the emergence of Kentucky in poultry remain few. This serotype shares virulence genes with other *Salmonella* serotypes which are implicated in the colonization and infection of the gastrointestinal tract, nevertheless, mechanisms differ between serotypes, and even within an individual serotype, underscoring how gene expression can significantly affect these functions. The discrepancy of Caco-2 cell invasion rate between Typhimurium and Kentucky is possibly due to discrimination in target host by serotype-related to genetic factors impacting host specificity including those linked with recognition and adhesion to intestinal surfaces and mechanisms of invasion of epithelial cells.

Our results correspond with previous findings that suggest infection by *Salmonella* can occur in a manner independent of T3SS [5–7, 39]. The *Salmonella* species appearing in the studies above were serotypes with broad host ranges. To our knowledge, this is the first study to replicate invasion, suggestive of T3SS-independent mechanisms, by a *Salmonella* serotype in a host not traditionally within its range. Due to the nature of invasion by Kentucky in the case of human colorectal epithelial cells, this possible alternative mechanism may exist as a minor invasive function with an ‘indiscriminative’ host range. *Salmonella* is capable of infecting diverse hosts and cells types, and an ‘indiscriminative’ mechanism may be evolutionarily beneficial to target a broad range of hosts.

The internalization into nonphagocytic cells of the intestinal epithelium is central to the pathogenesis of *Salmonella*, especially in relation to the maturation of infection. Most certainly it is viable to expect multiple mechanisms that perform overlapping functions to preserve the invasion process. Numerous invasion factors, such as Rck [40] and PagN [41, 42], have been studied that show manipulation of host signaling pathways to activate host actin nucleation to mediate the uptake of *Salmonella* into host cells [43, 44]. However, recently *Salmonella* Typhimurium was shown to manipulate additional signaling pathways leading to actomyosin-mediated contractility, suggesting a flexibility to target different cell types and/or subcellular regions [45, 46].

## Conclusions

Concurrent with the growth of the poultry industry and rising consumption of commercial poultry and related products, it is reasonable to be concerned with increased risk from poultry-associated foodborne related illnesses in the human population. *Salmonella* maintains a stable existence in commercial poultry due to its adapted, intimate relationship with its host [47–49]. This relationship, as research progresses, reveals a precise, dynamic communication that continues to evolve with variation of host and serotype, farm management practices, and flock vaccination [50, 51]. In this research, Kentucky was shown to actively invade a eukaryotic monolayer, though at a rate that was significantly lower than Typhimurium, a known human pathogen. Traditionally, Kentucky is not a zoonotic pathogen, and this result supports recent cases of the isolation of Kentucky from humans. Additionally, strains mutated for T3SS formation were able to initiate invasion of the eukaryotic monolayer to varying degrees based on strain, In the case of Kentucky, the mutated strain initiated invasion at a level that was not significantly different from the wild-type strain. A different result was observed for Typhimurium as the mutation significantly lowered the rate of invasion in comparison to the wild-type strain. Results from this study may provide the basis for additional research to identify factors delineating host specificity for strains of *Salmonella* as well as identify supplemental mechanisms for host invasion. Factors associated with invasion and host specificity will be critical to predicting how possible consequences of a shift in *Salmonella* population will impact the commercial food industry and public health.

## Abbreviations

*Cat*: Chloramphenicol acetyltransferase
Cm: Chloramphenicol
EMEM: Eagle’s Minimum Essential Medium
FRT: FLP recognition target
MOI: multiplicity of infection
NPIP: National Poultry Improvement Plan
SPI1: *Salmonella* Pathogenicity Island-1
T3SS: Type III secretion system
